# Supplementary material for: Evaluation of a Novel Mitochondrial Pan-Mucorales Marker for the Detection, Identification, Quantification, and Growth Stage Determination of Mucormycetes
Source: J Fungi (Basel). 2019 Oct 11;5(4):98. doi: 10.3390/jof5040098 (PMC6958370; doi:10.3390/jof5040098)
Supplement: Supplementary file 1 [file jof-05-00098-s001.pdf]

Supplementary materials

SUPPLEMENT TABLES

Table S 1: Reference strain set.

strain ID	species	substrate origin. country
CBS 102.48	*L. corymbifera*	moldy shoe. India
CBS 344.29	*R. microsporus var. chinensis*	- . ex USSR
CBS 388.34	*R. microsporus var. chinensis*	- . Japan
CBS 196.77	*R. microsporus var. rhizopodiformis*	herbal tea. USA
CBS 102277	*R. microsporus var. rhizopodiformis*	rhinocerebral infection. -
CBS 111233	*R. arrhizus*	- . -
CBS 118614	*R. arrhizus var. arrhizus*	palate. clinical. Turkey
CBS 109939	*R. arrhizus var. arrhizus*	human skin. Ontario
CBS 120808	*R. arrhizus*	sputum. France
CBS 124669	*R. microsporus*	soft palate. Greece
CBS 264.60	*R. arrhizus*	pig liver abscess Norway.
CBS 136236	*R. arrhizus*	- . -
CBS 136239	*R. arrhizus*	- . -
CBS 136240	*R. arrhizus*	- . -

(strain ID) - international culture collection, or internal laboratory number of the Division of Hygiene and Medical Microbiology; (CBS) - Centraalbureau voor Schimmelcultures Fungal Biodiversity Centre, Utrecht, The Netherlands; *(L.) - Lichtheimia; (M.) - Mucor; (R.) - Rhizopus; (Rh) – Rhizomucor;* (-) no information available at the CBS database.

Table S 2: Strain set.

strain ID	species identification[a]	origin (collection site)
15-09	*L. corymbifera*	clinical (sputum)
2-10	*L. ramosa*	clinical (biopsy)
7-10	*L. corymbifera*	clinical (tracheal secretion)
16-10	*L. corymbifera*	clinical (BAL)
30-10	*R. microsporus*	clinical (tissue sample)
31-10	*R. microsporus*	clinical (tissue sample)
39-10	*M. circinelloides*	clinical (swab)
41-10	*R. microsporus*	clinical (nasal swab)
63-10	*L. corymbifera*	clinical (swab)
75-10	*R. microsporus*	clinical (peritoneum)
92-10	*L. corymbifera*	clinical (hand swab)
1-11	*L. ramosa*	clinical (sputum)
11-11	*L. corymbifera*	clinical (culture isolate)
14-11	*R. microsporus*	clinical (culture isolate)
15-11	*L. corymbifera*	clinical (nasal swab)
24-11	*L. corymbifera*	clinical (BAL)
23-11	*L. corymbifera*	clinical (nose)
25-11	*L. corymbifera*	clinical (i-a biopsy)
63-11	*R. microsporus*	clinical (tissue sample)
71-11	*R. microsporus*	clinical (sputum)
4-12	*L. corymbifera*	clinical (skin lesion)
6-12	*L. corymbifera*	clinical (lung tissue)
33-12	*L. ramosa*	clinical (bronchial secretion)
42-12	*L. ramosa*	clinical (swab)
44-12	*R. arrhizus*	clinical (tracheal secretion)
88-12	*L. corymbifera*	clinical (nasal swab)
BOL-CR4	*R. arrhizus*	environmental (soil)
BPB-ES5	*R. arrhizus*	environmental (soil)
SPL-CR1	*M. circinelloides*	environmental (soil)
MAL-D1	*R. arrhizus*	environmental (soil)
MAL-D2	*Mucor sp.*[c]	environmental (soil)
MAL-D3	*M. circinelloides*	environmental (soil)
BOL-CR6	*M. circinelloides*	environmental (soil)
LUN-D1	*M. circinelloides*	environmental (soil)
LUN-D3	*R. arrhizus*	environmental (soil)
LLU-MA1	*R. arrhizus*	environmental (soil)
LLU-MA3	*R. arrhizus*	environmental (soil)
LLU-MA4	*R. arrhizus*	environmental (soil)
COP-D2	*M. circinelloides*	environmental (soil)
ROS-D1	*R. arrhizus*	environmental (soil)
FF17	*R. arrhizus*	clinical (BAL)
FF18	*R. arrhizus*	clinical (BAL)
HUM-D2	*M. circinelloides*	environmental (soil)
KOG-D3	*R. arrhizus*	environmental (soil)
KOG-D2	*M. circinelloides*	environmental (soil)
JBN-FR2	*M. circinelloides*	environmental (soil)
NEM-UK2	*M. circinelloides*	environmental (soil)
AS10	*L. corymbifera*	clinical (-)

AS15	*L. corymbifera*	clinical (-)
AS19	*L. ramosa*	clinical (-)
AS25	*L. corymbifera*	clinical (right eye lesion)
AS30	*L. corymbifera*	clinical (swab)
AS35	*L. ramosa*	clinical (-)
AS37	*R. microsporus var. chinensis*	clinical (-)
AS40	*R. microsporus*	clinical (-)
AS46	*L. corymbifera*	clinical (-)
AS47	*L. corymbifera*	clinical (-)
AS50	*L. ramosa*	clinical (-)
AS54	*L. corymbifera*	clinical (-)
AS61	*R. microsporus*	clinical (-)
AS64	*L. ramosa*	clinical (-)
AS66	*L. corymbifera*	clinical (-)
AS67	*L. corymbifera*	clinical (auditory canal)
AS74	*R. microsporus*	clinical (-)
AS76	*L. ramosa*	clinical (-)
AS81	*L. corymbifera*	clinical (-)
AS82	*R. microsporus*	clinical (-)
AS84	*M. circinelloides*	clinical (-)
AS88	*L. corymbifera*	clinical (lung tissue)
AS103	*L. corymbifera*	clinical (tracheal secretion)
AS109	*R. microsporus*	clinical (peritoneum pus)
AS110	*R. arrhizus*	clinical (-)
AS118	*L. corymbifera*	clinical (-)
AS119	*L. corymbifera*	clinical (-)
AS121	*L. corymbifera*	clinical (-)
F1	*L. corymbifera*	clinical (biopsy)
F2	*R. microsporus*	clinical (pleural tissue)
F7	*L. corymbifera*	clinical (BAL)
F13	*L. corymbifera*	clinical (-)
F19	*M. circinelloides*	clinical (nasal swab)
F20	*R. microsporus*	clinical (-)
F28	*L. corymbifera*	clinical (-)
F29	*L. corymbifera*	clinical (tracheal secretion)
F33	*R. microsporus*	clinical (peritoneum pus)
F50	*R. microsporus*	clinical (-)
F39	*L. corymbifera*	clinical (hand swab)
F83	*R. microsporus*	clinical (-)
CNM-CM4569	*M. circinelloides*	clinical (BAL)
CNM-CM5169	*M. circinelloides*	clinical (respiratory)
CNM-CM5170	*M. circinelloides*	clinical (respiratory)
CNM-CM5225	*M. circinelloides*	clinical (respiratory)
CNM-CM5364	*R. arrhizus*	clinical (-)
CNM-CM5368	*M. circinelloides*	clinical (abscess)
CNM-CM5389	*M. circinelloides*	clinical (optical exudate)
CNM-CM5542	*M. circinelloides*	clinical (skin biopsy)

[(a)] - Species identification based on ITS sequencing; [(b)] - second sample retrieved from same patient at a different time point; [(c)] - identification only possible to genus-level using the ITS sequencing; (*L*) - *Lichtheimia*; (*M.*) - *Mucor*; (*R.*) - *Rhizopus*; (BAL) -

bronchoalveolar lavage; (-)- no clinical information available from sample collection site; (i-a) - intra-abdominal; (strain ID) - international culture collection, or internallaboratory number of the Division of Hygiene and Medical Microbiology.

Table S 3: Single nucleotide polymorphisms (SNPs) found in the 276 bp rnl gene fragment. Nucleotide positions are according the consensus rnl sequence after alignment and trimming of sequences. Results are based on 82 isolates. The SNPs not covered by the real-time PCR-HRM are depicted in light grey.

	**positions of the SNPs found in the 276 bp rnl gene fragment**														
**species (nr. isolates)**	**113**	**156**	**177**	**185**	**188**	**193**	**196**	**197**	**201**	**202**	**203**	**204**	**207**	**208**	**272**
M. circinelloides (n=16)	G	A	G	G	C	T	G	G	indel	indel	A	A	C	C	A
R. arrhizus (n=13)	G	A	A	G	T	A	C	A	indel	indel	indel	T	T	G	A
L. ramosa (n=9)	G	G	A	A	C	A	C	A	indel	indel	indel	T	T	G	G
L. corymbifera (n=27)	G	G	A	A	C	A	C	A	indel	indel	indel	T	T	G	G
R. microsporus (n=17)	A	A	A	G	T	A	C	A	A	A	A	T	T	G	A

nr. number; bp. base pairs; *M. Mucor*; *R. Rhizopus*; *L. Lichtheimia*; indel. Insertion/deletion.

Table S 4: Results of rnl real-time PCR-HRM [(b)] and ITS sequencing [(a)]. 10^9 Mucorales isolates from our strain collection (Tables S1 and S2) were tested and identity was compared. For of rnl real-time PCR-HRM the following parameters are reported: identity, percentage confidence range [(c)], melting temperature (Tm) range, average and respective standard deviations are provided.

**strain nr.**	**ITS species ID[a]**	**HRM species ID[b]**	**confidence[c] [%]**	**Tm [°C]**	**Tm [°C]**
AS82	*R. microsporus*	*R. microsporus*	99.30	77.60	**77.79 ± 0.09**
71.11	*R. microsporus*	*R. microsporus*	99.80	77.70	
75-10	*R. microsporus*	*R. microsporus*	99.10	77.70	
AS109	*R. microsporus*	*R. microsporus*	99.00	77.70	
AS37	*R. microsporus*	*R. microsporus*	98.60	77.70	
AS40	*R. microsporus*	*R. microsporus*	99.50	77.70	
AS74	*R. microsporus*	*R. microsporus*	98.90	77.70	
CBS 102277	*R. microsporus*	*R. microsporus*	99.30	77.70	
CBS 344.29	*R. microsporus*	*R. microsporus*	99.90	77.70	
CBS 388.34	*R. microsporus*	*R. microsporus*	99.50	77.70	
F2	*R. microsporus*	*R. microsporus*	99.80	77.70	
CBS 631.82	*R. microsporus*	*R. microsporus*	100.00	77.75	
14-11	*R. microsporus*	*R. microsporus*	99.50	77.80	
41-10	*R. microsporus*	*R. microsporus*	99.80	77.80	
63-11	*R. microsporus*	*R. microsporus*	99.20	77.80	
CBS 124669	*R. microsporus*	*R. microsporus*	99.40	77.80	
CBS 196.77	*R. microsporus*	*R. microsporus*	99.40	77.80	
CBS 264.60	*R. microsporus*	*R. microsporus*	99.80	77.80	
F20	*R. microsporus*	*R. microsporus*	99.90	77.80	
30-10	*R. microsporus*	*R. microsporus*	98.70	77.90	
31-10	*R. microsporus*	*R. microsporus*	99.80	77.90	
AS61	*R. microsporus*	*R. microsporus*	99.00	77.90	
CBS 112586	*R. microsporus*	*R. microsporus*	98.10	77.90	
F33	*R. microsporus*	*R. microsporus*	98.90	77.90	
F50	*R. microsporus*	*R. microsporus*	98.80	77.90	
CBS 109939	*R. arrhizus*	*R. arrhizus*	97.10	77.90	**78.10 ± 0.11**
CBS 118614	*R. arrhizus*	*R. arrhizus*	98.60	77.90	
MAL-D2	*R. arrhizus*	*R. arrhizus*	97.50	77.90	
CBS 111233	*R. arrhizus*	*R. arrhizus*	99.70	78.00	
CBS 136236	*R. arrhizus*	*R. arrhizus*	99.80	78.00	
CBS 136239	*R. arrhizus*	*R. arrhizus*	99.90	78.00	
44.12	*R. arrhizus*	*R. arrhizus*	99.10	78.10	
AS110	*R. arrhizus*	*R. arrhizus*	99.20	78.10	
CBS 120808	*R. arrhizus*	*R. arrhizus*	99.20	78.10	
CBS 136237	*R. arrhizus*	*R. arrhizus*	99.00	78.10	
CBS 136240	*R. arrhizus*	*R. arrhizus*	98.10	78.10	
CM 5368	*R. arrhizus*	*R. arrhizus*	99.80	78.10	
LLU-MA1	*R. arrhizus*	*R. arrhizus*	99.80	78.10	
LLU-MA3	*R. arrhizus*	*R. arrhizus*	99.70	78.10	
LLU-MA4	*R. arrhizus*	*R. arrhizus*	99.20	78.10	
ROS-D1	*R. arrhizus*	*R. arrhizus*	99.60	78.10	
MAL-D1	*R. arrhizus*	*R. arrhizus*	97.50	78.15	
BOL-CR4	*R. arrhizus*	*R. arrhizus*	99.50	78.20	
BPB-ES5	*R. arrhizus*	*R. arrhizus*	99.80	78.20	
KOG-D3	*R. arrhizus*	*R. arrhizus*	99.30	78.20	
LUN-D3	*R. arrhizus*	*R. arrhizus*	98.90	78.20	

FF17	*R. arrhizus*	*R. arrhizus*	99.60	78.30	
FF18	*R. arrhizus*	*R. arrhizus*	99.60	78.30	
15-09	*L. corymbifera*	*L. corymbifera* species complex	96.90	78.40	**78.54 ± 0.07**
63-10	*L. corymbifera*	*L. corymbifera* species complex	99.30	78.40	
CBS 102.48	*L. corymbifera*	*L. corymbifera* species complex	99.70	78.40	
92-10	*L. corymbifera*	*L. corymbifera* species complex	99.50	78.50	
F28	*L. corymbifera*	*L. corymbifera* species complex	99.60	78.50	
25-11	*L. corymbifera*	*L. corymbifera* species complex	99.70	78.50	
AS54	*L. corymbifera*	*L. corymbifera* species complex	99.70	78.50	
AS67	*L. corymbifera*	*L. corymbifera* species complex	99.70	78.50	
23-11	*L. corymbifera*	*L. corymbifera* species complex	99.80	78.50	
AS66	*L. corymbifera*	*L. corymbifera* species complex	99.80	78.50	
AS88	*L. corymbifera*	*L. corymbifera* species complex	99.80	78.50	
15-11	*L. corymbifera*	*L. corymbifera* species complex	99.90	78.50	
6-12	*L. corymbifera*	*L. corymbifera* species complex	99.90	78.50	
AS10	*L. corymbifera*	*L. corymbifera* species complex	99.90	78.50	
F13	*L. corymbifera*	*L. corymbifera* species complex	99.90	78.50	
F7	*L. corymbifera*	*L. corymbifera* species complex	99.90	78.50	
4-12	*L. corymbifera*	*L. corymbifera* species complex	100.00	78.50	
7-10	*L. corymbifera*	*L. corymbifera* species complex	99.90	78.55	
88-12	*L. corymbifera*	*L. corymbifera* species complex	99.20	78.60	
AS30	*L. corymbifera*	*L. corymbifera* species complex	99.30	78.60	
AS47	*L. corymbifera*	*L. corymbifera* species complex	99.40	78.60	
AS15	*L. corymbifera*	*L. corymbifera* species complex	99.50	78.60	
24-11	*L. corymbifera*	*L. corymbifera* species complex	99.70	78.60	
AS81	*L. corymbifera*	*L. corymbifera* species complex	99.70	78.60	
AS121	*L. corymbifera*	*L. corymbifera* species complex	99.80	78.60	
16-10	*L. corymbifera*	*L. corymbifera* species complex	99.90	78.60	
AS103	*L. corymbifera*	*L. corymbifera* species complex	99.90	78.60	
AS118	*L. corymbifera*	*L. corymbifera* species complex	99.90	78.60	
AS119	*L. corymbifera*	*L. corymbifera* species complex	99.90	78.60	
F29	*L. corymbifera*	*L. corymbifera* species complex	100.00	78.60	
AS46	*L. corymbifera*	*L. corymbifera* species complex	98.60	78.65	
AS32	*L. ramosa*	*L. corymbifera* species complex	99.30	78.40	**78.55 ± 0.09**
AS76	*L. ramosa*	*L. corymbifera* species complex	99.80	78.40	
2-10	*L. ramosa*	*L. corymbifera* species complex	99.30	78.50	
AS19	*L. ramosa*	*L. corymbifera* species complex	100.00	78.50	
AS25	*L. ramosa*	*L. corymbifera* species complex	99.90	78.50	
F1	*L. ramosa*	*L. corymbifera* species complex	99.40	78.50	
1-11	*L. ramosa*	*L. corymbifera* species complex	99.70	78.60	
11.11	*L. ramosa*	*L. corymbifera* species complex	99.80	78.60	
33-12	*L. ramosa*	*L. corymbifera* species complex	99.60	78.60	
42.12	*L. ramosa*	*L. corymbifera* species complex	99.60	78.60	
AS35	*L. ramosa*	*L. corymbifera* species complex	99.80	78.60	
AS64	*L. ramosa*	*L. corymbifera* species complex	99.80	78.60	
AS50	*L. ramosa*	*L. corymbifera* species complex	99.10	78.70	
MAL-D3	*M. circinelloides*	*M. circinelloides*	100.00	79.00	**79.09 ± 0.08**
39.10	*M. circinelloides*	*M. circinelloides*	99.80	79.00	
CM5364	*M. circinelloides*	*M. circinelloides*	99.70	79.00	
CM5389	*M. circinelloides*	*M. circinelloides*	99.70	79.00	

COP-D2	*M. circinelloides*	M. circinelloides	99.50	79.00
HUM-D2	M. circinelloides	M. circinelloides	99.20	79.00
KOG-D2	*M. circinelloides*	*M. circinelloides*	99.50	79.00
CM5542	*M. circinelloides*	*M. circinelloides*	99.60	79.10
JBN-FR2	*M. circinelloides*	*M. circinelloides*	99.70	79.10
SPL-CR1	*M. circinelloides*	*M. circinelloides*	97.60	79.10
AS84	*M. circinelloides*	*M. circinelloides*	99.90	79.15
CM5225	*M. circinelloides*	*M. circinelloides*	99.30	79.15
F19	*M. circinelloides*	*M. circinelloides*	99.80	79.15
LUN-D1	*M. circinelloides*	*M. circinelloides*	99.30	79.15
CM4569	*M. circinelloides*	*M. circinelloides*	99.70	79.20
CM5170	*M. circinelloides*	*M. circinelloides*	99.90	79.20
BOL-CR6	*M. circinelloides*	*M. circinelloides*	99.90	79.22

Table S 5: Standard deviations of standard curves displayed in Figure 6.

SD Values | *Rhizopus arrhizus*

Conidia concentration	Brain	Kidney	Lung	Liver	Spleen
10^8	23.77 ± 0.85	26.37 ± 0.90	24.15 ± 0.43	28.21 ± 0.49	27.77 ± 1.07
10^7	27.32 ± 0.07	27.65 ± 1.13	26.94 ± 0.69	29.86 ± 1.64	28.58 ± 1.59
10^6	29.90 ± 0.24	31.77 ± 0.38	29.95 ± 0.71	33.69 ± 1.70	32.10 ± 1.34
10^5	32.60 ± 0.59	32.83 ± 1.55	32.39 ± 2.03	34.51 ± 0.72	34.18 ± 0.93
10^4	34.68 ± 1.76	35.49 ±1.23	33.99 ± 2.08	35.86 ± 0.92	34.86 ± 0.83

SD Values | *Lichtheimia corymbifera*

Conidia concentration	Brain	Kidney	Lung	Liver	Spleen
10^8	28.20 ± 2.19	28.89 ± 0.81	27.65 ± 1.99	30.01 ± 0.66	27.03 ± 0.58
10^7	31.08 ± 1.76	32.19 ± 1.10	31.12 ± 1.14	32.26 ± 0.95	31.30 ± 1.09
10^6	33.10 ± 0.77	33.56 ± 0.79	33.17 ±0.56	35.34 ± 0.36	35.08 ± 0.94
10^5	35.12 ± 1.25	34.93 ±1.37	34.75 ± 0.78	35.77 ± 0.76	35.75 ± 1.45
10^4	36.05 ± 2.07	35.99 ± 0.64	36.57 ± 0.90	36.41 ± 0.36	36.20 ± 1.73

SUPPLEMENT FIGURES

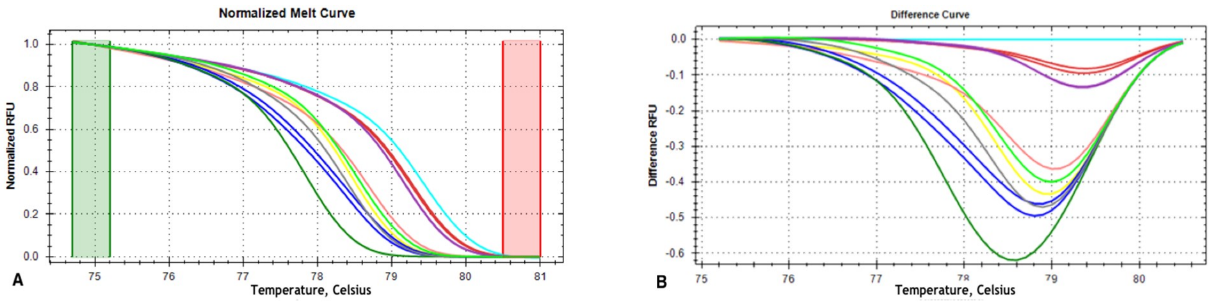

C

species	cluster number[a]	confidence[b] [%]	T_m [°C] duplicate average
Mucor racemosus	cluster 5	94.00	79.30
Phycomyces blakesleeanus	cluster 6	94.50	79.15
Cunninghamella bertholletiae	cluster 7	89.10	78.60
Syncephalastrum racemosum	cluster 8	93.70	78.40
Rhizomucor pusillus	cluster 9	99.20	78.35

Legend:
positive controls: *L. corymbifera*, *M. circinelloides*, *R. arrhizus*, *R. microsporus*
tested species: *C. bertholletiae*, *M. racemosus*, *P. blakesleeanus*, *R. pusillus*, *S. racemosum*

Figure S 1: Results for the HRM analysis of non-targeted Mucorales species (*Mucor racemosus, Phycomyces blakesleeanus, Cunninghamella bertholletiae and Rhizomucor pusillus*) are shown together with the melt profiles of species of interest (*Mucor circinelloides, Lichtheimia corymbifera species complex, Rhizopus arrhizus,* and *Rhizopus microsporus*). The experiments were performed to rule out overlapping melt curve profile with other Mucorales species. Melt profiles were computed with Precision Melt Analysis software v 1.2. **A** the areas shaded in green and in red are the pre- and post-melt regions, respectively; the raw melt curves are depicted, before normalization, showing the melt profiles for the positive Mucorales controls and the five other Mucorales, all in different colors; in **B** different shapes of melt curves and subsequently different cluster assignment; *M. circinelloides* was set as reference for automatic clustering. **C** shows percent confidence for automatic clustering of the five other Mucorales and respective melt temperatures.

Footer

[a] cluster numbers were randomly named according to the order of appearance in the HRM analysis;

[b] assignment made by Precision Melt Analysis software v 1.2 and is expressed in percentages; Tm .melting temperatures.

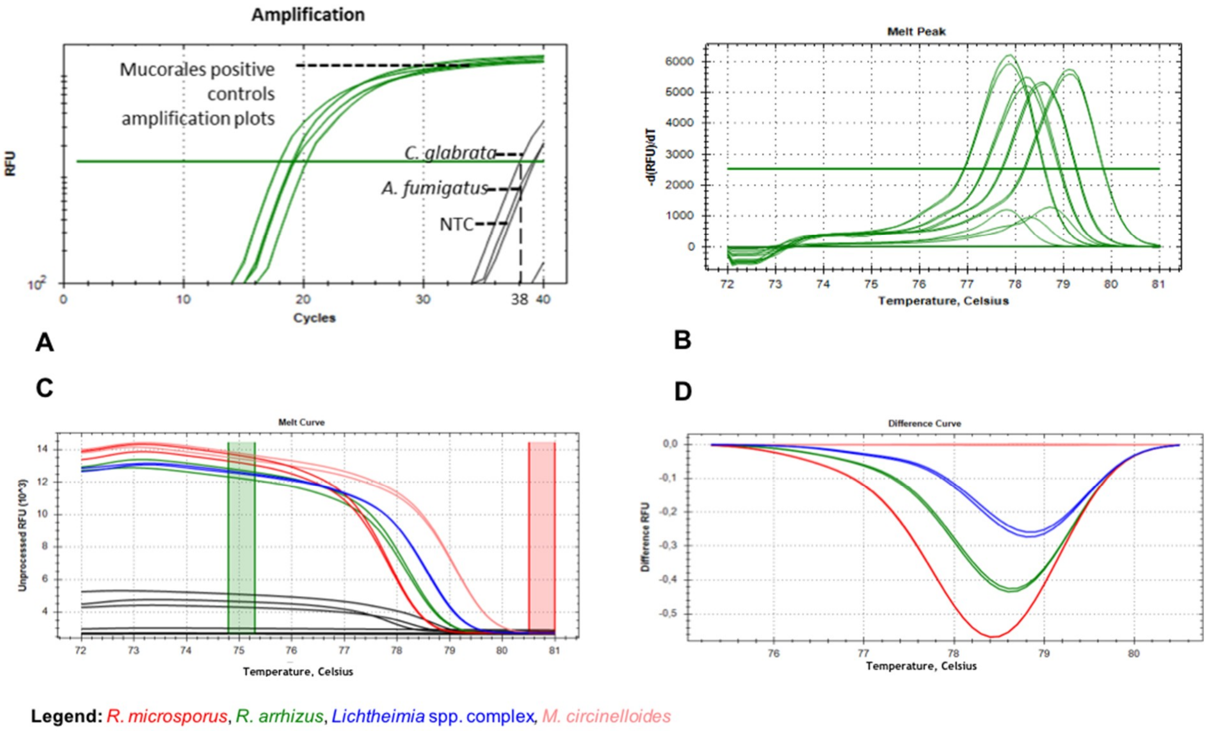

Figure S 2:Cross reactivity testing of human pathogenic fungi. (A) Amplification plots for four positive control Mucorales DNAs and DNA samples of fungal pathogens other than Mucorales species; one replicate of the *Candida glabrata* isolate shows amplification after 38 cycles, while one of the 2 replicates of the *Aspergillus fumigatus* sample and one of the no template control (NTC) shows the same after 39 cycles; **B)** Melt peaks for the same samples as A; the peak for *Rhizopus microsporus* is depicted in dark green, for *Rhizopus arrhizus* in salmon, *Lichtheimia corymbifera* species complex in light green and *Mucor circinelloides* is colored in light blue; the other fungal organisms exhibit no melt peak and these plots are colored in black; **C)** and **D)** The results observed for the high resolution melt analysis step processed by using the Precision Melt Analysis™ software v 1.2; in **C)** The areas shaded in green and in red are the pre- and post-melt regions, respectively; the raw melt curves are depicted, before normalization, showing the melt curves generated for the positive Mucorales controls in colors and the black lines shown in the graph represent the plots for the non-Mucorales organisms, for which no melt curves are observed; **D)** Depicts a typical difference curve for the four Mucorales positive controls analyzed, by using as reference cluster the melt profile generated for *M. circinelloides*.

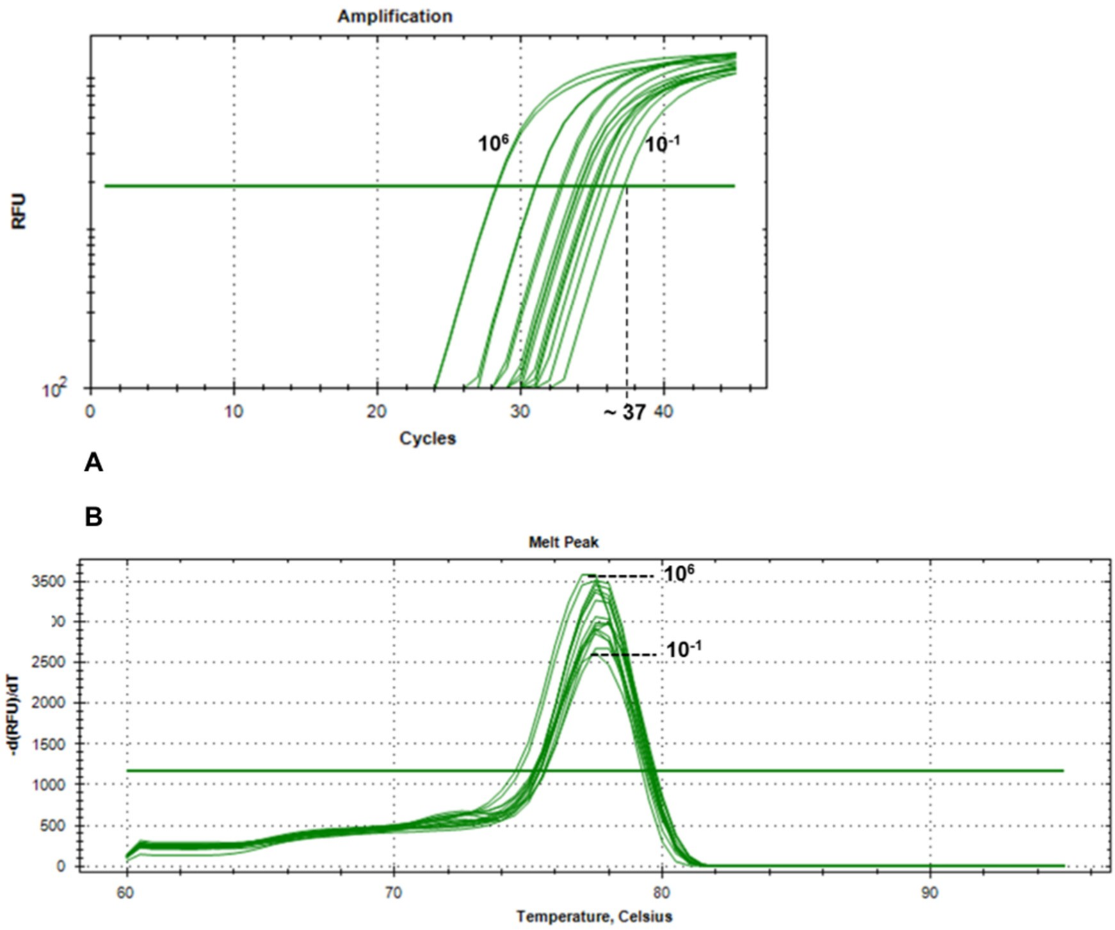

Figure S 3: Amplification plots and respective melt peaks obtained for the serial dilution of 10^6 to 10^{-1} conidia spiked in human whole blood samples for defining limit of detection (LoD). The strain *R. arrhizus* KOG-D3 was used for the spiking experiment. In **A** the amplification plots show a positive amplification until the lowest tested concentration (0.1 conidia/PCR reaction), at an average C_t of 36.98**. B** Respective melt peaks are show also to be linear until the same concentration.

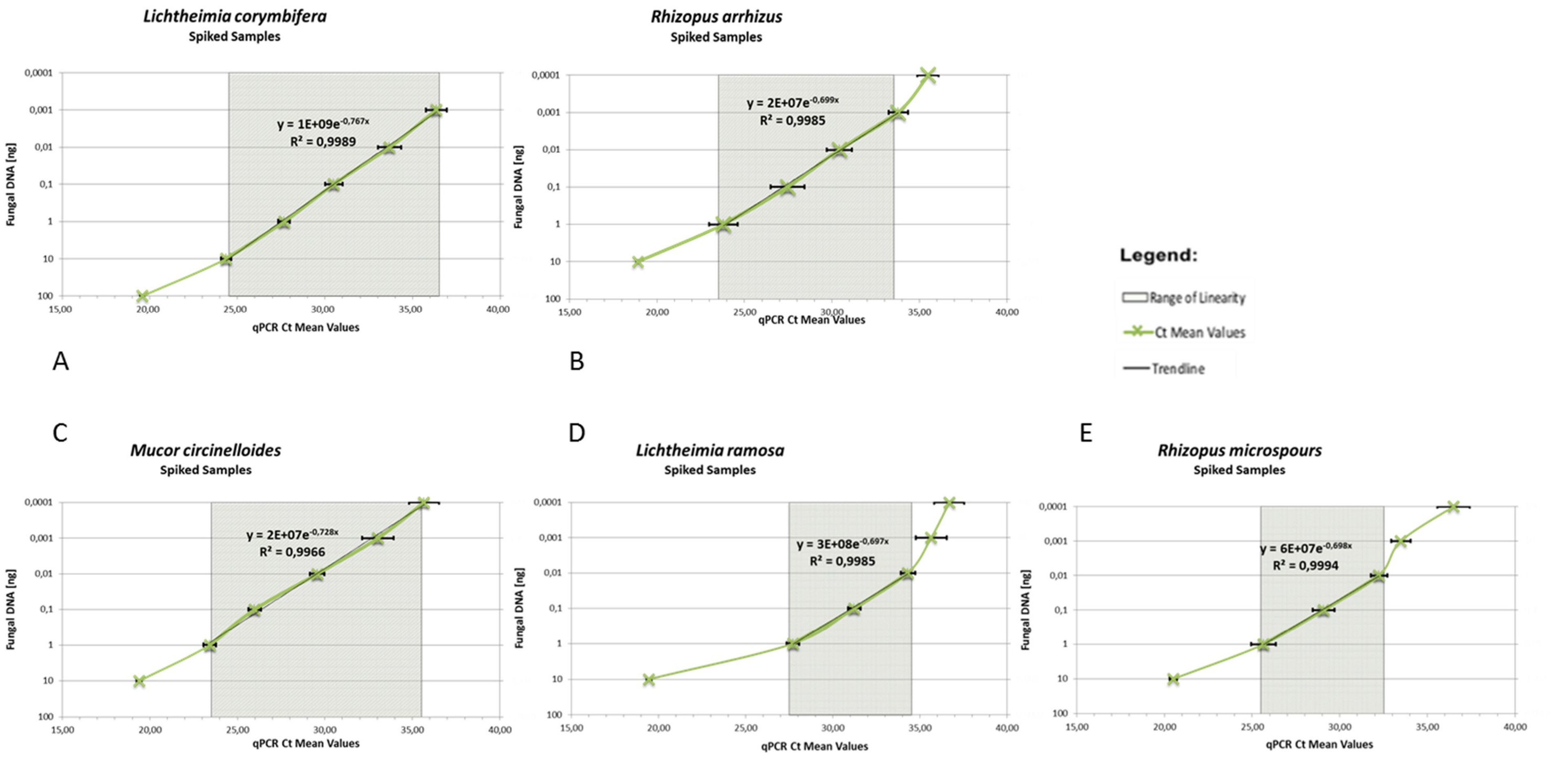

Figure S 4: Limit of detection (LoD) of rnl real-time PCR-HRM for EDTA-treated human whole blood samples spiked with mucoralean DNA. Range of linearity, trend line with coefficient of determination and linear equation, as well as C_t values for the corresponding dilution steps are shown for **A)** *L. corymbifera*, **B)** *R. arrhizus*, **C)** *M. circinelloides*, **D)** *L. ramosa*, and **E)** *R. microsporus*. Green shaded boxes indicated the level of linearity of the curve that enable the values R^2 to be calculated. LOD is marked as the right margin of the green box.

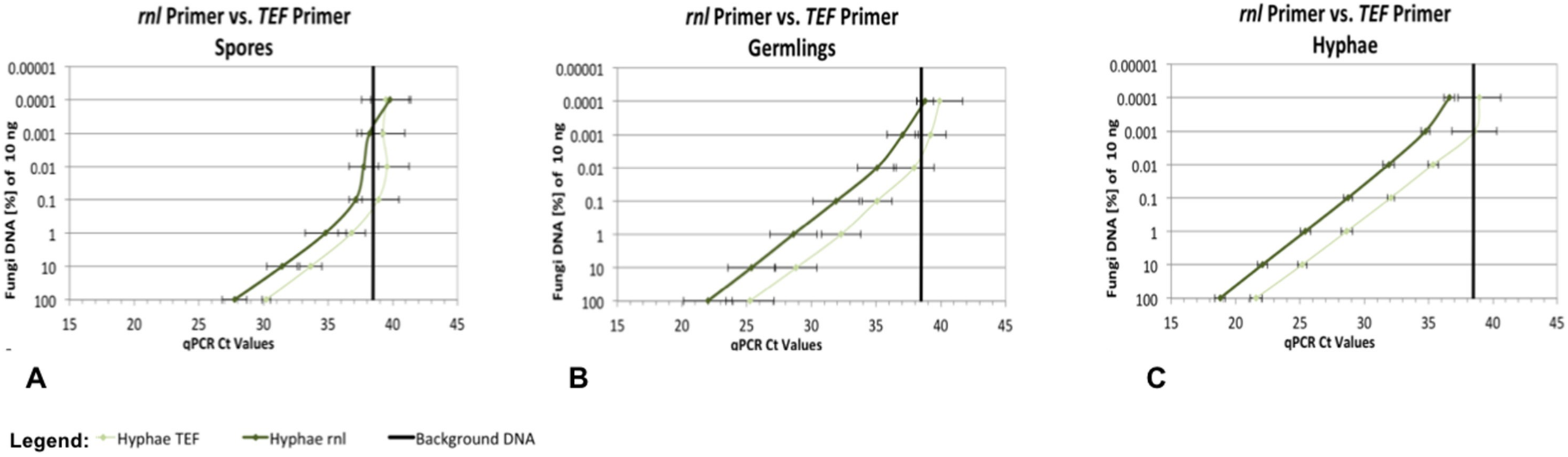

Figure S 3: Divergence in real-time PCR C_t values observed by rnl marker and tef housekeeping gene marker. Growth stages **A)** spores, **B)** germlings and **C)** hyphae were determined using a ten-fold dilution series of fungal DNA [% of total DNA concentration of 10 ng/µl] in EDTA-treated human whole blood spiked samples. Ranges and mean values are given as overall results for the species *Lichtheimia* spp. complex, *R. arrhizus*, *R. microsporus*, and *M. circinelloides* .
